# Decreased *CDKL2* Expression in Clear Cell Renal Cell Carcinoma Predicts Worse Overall Survival

**DOI:** 10.3389/fmolb.2021.657672

**Published:** 2022-01-13

**Authors:** Zhan Chen, Yan Lv, Lu He, Shunli Wu, Zhuang Wu

**Affiliations:** Department of Urology, CR & WISCO General Hospital, Wuhan University of Science and Technology, Wuhan, China

**Keywords:** Cdkl2, clear cell renal cell carcinoma, immune infiltrates, biomarker, overall survival

## Abstract

**Background:** Clear cell renal cell carcinoma (ccRCC) is the most frequent and lethal type of kidney cancer. Although differential expression of cyclin-dependent kinase-like 2 (*CDKL2*) has been reported to be associated with tumor progression in other cancers, its prognostic value, and potential mechanism in patients with ccRCC still remain unknown.

**Methods:** Gene expression analysis was conducted using The Cancer Genome Atlas (TCGA), Gene Expression Omnibus, and International Cancer Genome Consortium databases. Further, clinicopathologic analysis; Kaplan–Meier survival analysis; weighted gene co-expression network analysis; gene set enrichment analysis; gene ontology enrichment; methylation; and immune infiltration analyses were performed using TCGA-kidney renal clear cell carcinoma profiles. CDKL2 translational levels were analyzed using The Human Protein Atlas database.

**Results:** *CDKL2* expression was decreased in ccRCC samples retrieved from the four databases. Gender, survival status, histologic grade, clinical stage, TNM classification, and tumor status were closely related to *CDKL2* expression. In addition, *CDKL2* downregulation was an independent prognostic factor for poor prognosis in multivariate analysis. Enrichment analyses using multiple tests revealed that *CDKL2* is not just closely related to immune response but this association is highly correlated as well. Further, we found that *CDKL2* expression was significantly correlated with the infiltration levels of T cell CD4 memory resting; monocytes; macrophages M0, M1, and M2; dendritic cells resting; mast cells resting; plasma cells; T cell CD8; and T cell regulatory.

**Conclusion:** This is the first report to study the expression of *CDKL2* in ccRCC, wherein we suggest that decreased *CDKL2* expression is closely correlated with poor prognosis in ccRCC. We consider that *CDKL2* is a novel and potential prognostic biomarker associated with immune infiltrates in ccRCC.

## Introduction

In 2019, kidney cancer is the sixth and eighth most commonly diagnosed cancers in men and women, respectively. There were 73,820 Americans diagnosed with carcinoma of the kidney and renal pelvis; of them, an estimated 14,770 would die of the disease in 2019 (Siegel et al., 2019). Renal cell carcinoma (RCC) is the most frequent subtype of kidney cancer that accounts for over 90% of all renal malignancies (Lipworth et al., 2016). Further, clear cell renal cell carcinoma (ccRCC) accounts for approximately 70% of all cases of RCC. ccRCC has an extremely poor prognosis owing to its considerably high rate of metastasis, vascular invasion, recurrence, and mortality. Moreover, it easily develops resistance to radiotherapy and chemotherapy (Rini et al., 2009). Currently, standard surgical resection is widely used for effective treatment of localized tumor. However, approximately 30% of these patients are diagnosed with local invasion or distant metastasis. Moreover, about one third of them with local RCC experience tumor recurrence after nephrectomy (Escudier et al., 2016). Hence, it is extremely crucial to explore novel and more reliable and effective biomarkers that can reveal the molecular mechanisms of tumor progression, thereby improving prognosis of ccRCC and intervention benefit in patients.

*CDKL2* encodes the cyclin-dependent kinase-like 2 (CDKL2) protein, also known as P56 and KKIAMRE. CDKL2 is a member of the CDC2-related serine/threonine protein kinase family and is similar to mitogen-activated protein kinase (MAPK) family based on genetics and biochemical studies. (Malumbres et al., 1996; Yeh et al., 2013; Zhou et al., 2019). It mainly accumulates in the cytoplasm, while having a relatively lower level in the nucleus. Several studies have shown that CDKL2 expressed in the nervous system plays a role in cognitive functions, emotion, and neurological disorders (Gomi et al., 1999; Sassa et al., 2000; Sassa et al., 2004; Gomi et al., 2010; Canning et al., 2018). In the Genecards database, papillary serous adenocarcinoma is included among the diseases associated with *CDKL2.* Moreover, recent studies have indicated that *CDKL2* is associated with malignant tumors, such as prostate cancer (Rubicz et al., 2019), breast cancer (Li et al., 2014; Lindqvist et al., 2014), hepatocellular carcinoma (HCC) (Shen et al., 2012; Zhou et al., 2019), glioma (Yi et al., 2020), and gastric cancer (Fang et al., 2018).

In this study, we conducted an integrated bioinformatic investigation in kidney renal clear cell carcinoma (KIRC) cohort from the data available at The Cancer Genome Atlas (TCGA). We analyzed the expression of *CDKL2* with respect to the clinicopathological features, prognostic value, and potential mechanism.

## Materials and Methods

### *CDKL2* Expression Level Using TIMER2.0 Web Tool

TIMER2.0 is the latest web resource that includes samples of various cancer types accessible in the TCGA cohort. This tool can be used for systematic analysis of immune infiltrate signatures in different cancer types (https://cistrome.shinyapps.io/timer/) (Li et al., 2020). We used the tool’s Gene_DE in Exploration Module to evaluate the *CDKL2* differential expression between normal tissues and various tumor tissues. Further, we used Gene_Outcome Module to investigate the clinical relevance of *CDKL2* expression levels across different cancer types. This module uses the Cox proportional hazard model to evaluate the outcome association of gene expression.

### Data Acquisition From Several Databases

We retrieved standardized gene expression data (611 cases, N = 72, T = 539, Workflow Type: HTSeq-FPKM) and their relevant clinical data profiles from the TCGA database for KIRC projects in January 2020. Next, we used the merge script of Perl 5.26.1 language to separately merge the RNA-seq and clinical data files into single matrix file. Ensemble gene IDs were transformed to official gene symbols by Perl language. To identify *CDKL2* mRNA expression between tumor (N = 539) and non-cancerous kidney tissue (N = 72) samples, *limma* packages (Ritchie et al., 2015) were used. Further, we obtained *CDKL2* expression profiles of GSE40435 (N = 101, T = 101) (Wozniak et al., 2013) and GSE53757 (N = 72, T = 72) (von Roemeling et al., 2014) from Gene Expression Omnibus (GEO) database. In addition, RCC data from International Cancer Genome Consortium (ICGC) were retrieved and *CDKL2* in 45 normal and 91 tumor tissue samples were compared to identify differential expression. To identify translational levels of *CDKL2*, we obtained immunohistochemistry sections of ccRCC and normal tissues from The Human Protein Atlas database (HPA) (https://www.proteinatlas.org/) (Uhlen et al., 2017). Furthermore, E-MTAB-3267 dataset in ArrayExpress database ((https://www.ebi.ac.uk/arrayexpress/) was used to validation the result, there 59 samples in total, 6 sample was normal tissues and have no progression-free survival (PFS) information, the rest 53 samples were clear cell carcinoma pathological type.

### Evaluation of Patient Characteristics

We obtained *CDKL2* expression samples from TCGA to test diagnostic value of *CDKL2*. Receiver operating characteristic (ROC) curve was generated for estimating a biomarker that predicts the prognostic survival of patients. Based on multivariate logistic regression, a nomogram was accessed using “rms” (Harrell, 2020) and “foreign” (R Core Team, 2018) packages in R. Additionally, we evaluated the concordance index (c-index) and compared the nomogram-predicted estimates with Kaplan–Meier estimates of survival probability.

### Co-Expression Analysis of *CDKL2* Using WGCNA

To investigate the potential genes co-expressed with *CDKL2*, the top 1,000 remarkable positively and negatively correlated genes were extracted from TCGA-KIRC database and examined one by one using Pearson correlation test. *p* < 0.05 was chosen as the threshold criterion. We divided the 2000 extracted genes into different gene modules using WGCNA package (Langfelder and Horvath, 2008) of R (min Module Size = 30). We aimed to identify the relationship between the co-expressed gene module and clinical characteristics, including futime (survival time), fustat (survival status), gender, histologic grade, clinical stage, and tumor status.

### Analysis of *CDKL2* Methylation in ccRCC

UALCAN (Chandrashekar et al., 2017), a web tool based on TCGA cohort, was used to identify the methylation levels of *CDKL2* in ccRCC and normal tissue samples. We browsed the TCGA-KIRC database at LinkedOmics (Vasaikar et al., 2018) to analyze the co-expression of genes induced by *CDKL2* methylation using Pearson correlation test. Volcano map and heatmaps were generated to show the results. For further analysis, MethSurv (Modhukur et al., 2018) was chosen to perform survival analysis based on single CpG methylation of *CDKL2*.

### Biological Processes and KEGG Analysis of Gene Modules and *CDKL2* Methylation-Related Genes

Genes in the WGCNA key modules and *CDKL2* methylation-related genes were subjected to Gene Ontology and Kyoto Encyclopedia of Genes and Genomes (KEGG) pathway analyses with DAVID (Huang et al., 2009a; Huang et al., 2009b) 6.8, respectively. *p* < 0.05 was chosen as the criterion. Oncobox (Sorokin et al., 2021) was a free and convenient webtool that can identify functional roles of the pathway components and applied it to annotate 3,044 human molecular pathways extracted from the Biocarta, Reactome, KEGG, Qiagen Pathway Central, NCI, and Human CYC databases and including 9,022 gene products.

### Gene Set Enrichment Analysis of *CDKL2*

We used the computational method GSEA that determines statistical significance of a priori-defined set of genes and existence of concordant differences between two biological states (Mootha et al., 2003; Subramanian et al., 2005). In this study, we set the gene set permutations for each analysis a thousand times. *CDKL2* expression level was used as phenotype label. The gene sets “kegg (v7.1),” “bp (v7.1),” and “hallmark (v7.1)” were retrieved from the Molecular Signatures Database; they were subjected to GSEA 4.0.3 to analyze the potential enrichment pathways and biological processes (BP). Additionally, Normalized Enrichment Scores (NESs), nom *p*-value, and FDR q-value in GSEA were generated to sort the enriched pathways into two phenotypes. In our study, gene sets with a nom *p*-value < 0.05 and an FDR q-value < 0.25 were considered remarkably enriched.

### Analysis of the Relative Abundance of Tumor-Infiltrating Immune Cells

To assess the relative correlation between *CDKL2* expression and TIICs, we used CIBERSORT (http://cibersort.stanford.edu/), which is a deconvolution algorithm based on gene expression. This method aided in characterizing the cell composition of complex tissues (Newman et al., 2015). Immune responses of 22 TIICs were used to evaluate the relationship between high and low *CDKL2* expression groups using the CIBERSORT method. Further, we calculated the *p*-value of each sample according to the deconvolution algorithm.

### Statistical Analysis

Statistical analyses were conducted using R software (v.3.6.1) and SPSS 22. Median value of *CDKL2* expression was chosen as the cut-off value. Clinicopathological features associated with high and low *CDKL2* expression groups were analyzed using the chi-square and Fisher exact tests. Additionally, correlation between *CDKL2* expression and clinicopathological features was analyzed using Wilcoxon signed-rank test. Kruskal–Wallis test was used for multigroup comparison (such as tumor stage, clinical stage, and pathological grade). To estimate the influence of *CDKL2* expression on survival and clinicopathological factors, overall survival (OS) in TCGA patients was assessed using Cox regression and the Kaplan–Meier method. *p* < 0.05 was considered significant.

## Results

### Differential *CDKL2* Expression in ccRCC Compared to Normal Kidney Tissue Samples

**FIGURE 1:**
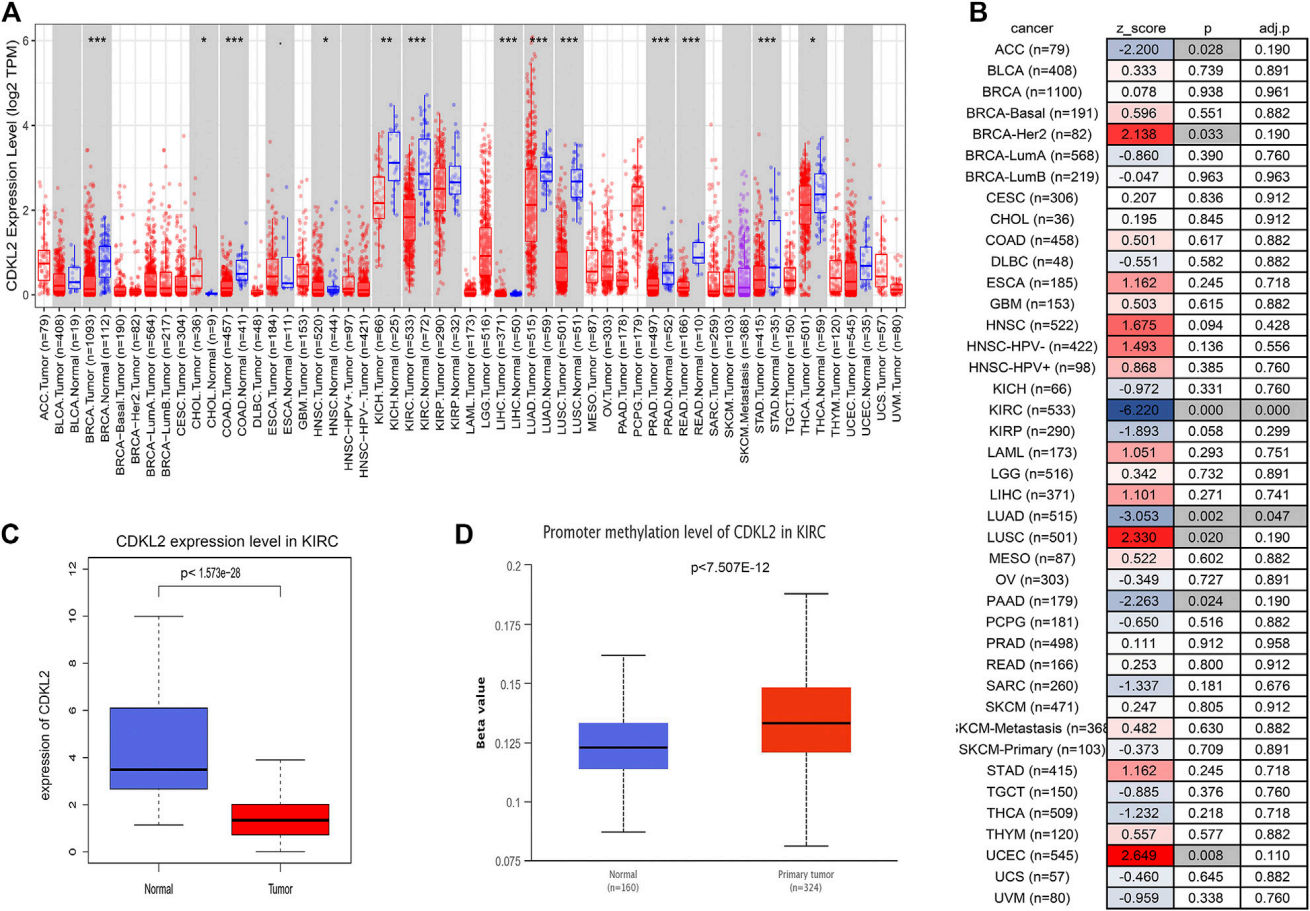
CDKL2 expression levels in different types of cancers in TCGA. **(A)** Human CDKL2 expression levels in different tumor types from TIMER tool. **(B)** Heatmap was drawn to show the normalized coefficient of the CDKL2 in Cox model from TIMER outcome module. **(C)** The KIRC database verified that CDKL2 gene expression was significantly downregulated in ccRCC (*n* = 539) compared with normal kidney tissues (*n* = 72), **(D)**, The methylation level of CDKL2 in the normal kidney tissues and ccRCC tissues. *p < 0.05, **p < 0.01, ***p < 0.001.

We estimated *CDKL2* mRNA levels in a variety of human tumors using the TIMER2.0 web tool. As shown in Figures 1A,B, *CDKL2* mRNA levels in ccRCC tissue samples were lower than those in normal kidney tissue samples. Also, *CDKL2* was expressed differently in tumor tissues of other organs and normal tissues, such as liver, lung, breast, brain, colon, rectum, uterus, bile duct, pancreas, stomach, and thyroid. Only in cholangiocarcinoma (CHOL) and pheochromocytoma and paraganglioma (PCPG), *CDKL2* expression is up-regulated in tumor tissues. In the TIMER2.0 Gene_Outcome Module, we found that *CDKL2* was most strongly associated with a decreased risk of OS (*p* < 0.05, z < 0). For further analysis, we assessed the *CDKL2* mRNA level in ccRCC using TCGA data, where *CDKL2* was downregulated in ccRCC samples as compared to normal samples (Figure 1C).

### Relationship Between *CDKL2* and Clinicopathological Features in TCGA Cohort

**FIGURE 2:**
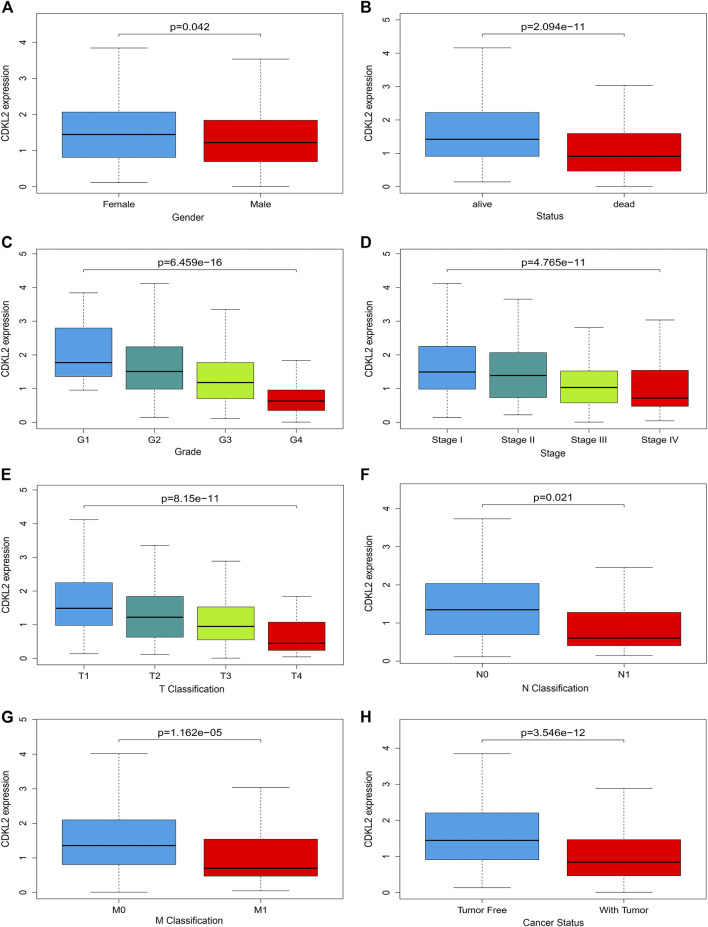
Associations between CDKL2 gene expressions and clinicopathological parameters in ccRCC. **(A)** Gender, **(B)** Patients lived status, **(C)** Tumor grade, **(D)** TNM stage, **(E)** T classification, **(F)** N classification (Lymph node metastasis), **(G)** M classification (Distant metastasis), **(H)** Cancer status (with tumor *vs.*tumor free).

Clinical datasets of 537 patients with ccRCC were retrieved from TCGA-KIRC database. Detailed clinicopathological information is shown in Table 1. Subsequently, all cases were divided into high and low *CDKL2* expression groups based on the median value of *CDKL2* expression. Gender, survival status, histologic grade, clinical stage, cancer status, and T, N, and M classification were significantly correlated with *CDKL2* expression (Figure 2). Correlation between *CDKL2* expression and the clinicopathologic variables of ccRCC is summarized in Table 2. Consistent with the result in Figure 2, *CDKL2* expression level was highly correlated with gender, survival status, histologic grade, clinical stage, T and M classification, and tumor status.

**TABLE 1:** TCGA-KIRC patient characteristics.

Clinical characteristics	Total (N = 537)	Percent (%)
Age (y)		
>60	271	50.5
≤60	266	49.5
Gender		
Female	191	35.6
Male	346	64.4
Survival status		
Alive	361	67.2
Dead	176	32.8
Histologic grade		
Grade Ⅰ	14	2.6
Grade Ⅱ	230	42.8
Grade Ⅲ	207	38.5
Grade Ⅳ	78	14.5
Grade X	8	1.5
Clinical stage		
Stage Ⅰ	269	50.1
Stage Ⅱ	57	10.6
Stage Ⅲ	125	23.3
Stage Ⅳ	83	15.5
Stage X	3	0.5
N classification		
N0	240	44.7
N1	17	3.2
NX	280	52.1
M classification		
M0	446	83.1
M1	81	15.1
MX	10	1.8
Tumor status		
With tumor	141	26.3
Tumor free	361	67.2
Not available	35	6.5

**TABLE 2:** Relationship between the clinicopathological characteristics and CDKL2 expression.

Parameter	Variable	N (530)	CDKL2 mRNA expression		χ2	P
			High (N = 265)	Low (N = 265)		
Age (y)	>60	266	137	129	0.483	0.487
	≤60	264	128	136		
Gender	Female	186	108	78	7.455	0.006
	Male	344	157	187		
Survival status	Alive	364	210	154	27.507	0.000
	Dead	166	55	111		
Histologic grade	GradeⅠ	14	11	3	51.653	0.000
	GradeⅡ	227	141	86		
	GradeⅢ	206	95	111		
	GradeⅣ	75	13	62		
	Grade X	8	5	3		
Clinical stage	StageⅠ	265	163	102	35.354	0.000
	StageⅡ	57	30	27		
	StageⅢ	123	43	80		
	Stage Ⅳ	82	29	53		
	Stage X	3	0	3		
T classification	T1	267	163	104	59.727	0.000
	T2	66	30	36		
	T3	160	43	117		
	T4	37	29	8		
N classification	N0	239	121	118	4.129	0.127
	N1	16	4	12		
	NX	275	140	135		
M classification	M0	420	220	200	7.283	0.026
	M1	78	28	50		
	MX	32	17	15		
Tumor status	With tumor	138	43	95	28.825	0.000
	Tumor free	358	207	151		
	Unknow	34	15	19		

### Low *CDKL2* Expression Associated With Poor OS in Patients With ccRCC

**FIGURE 3:**
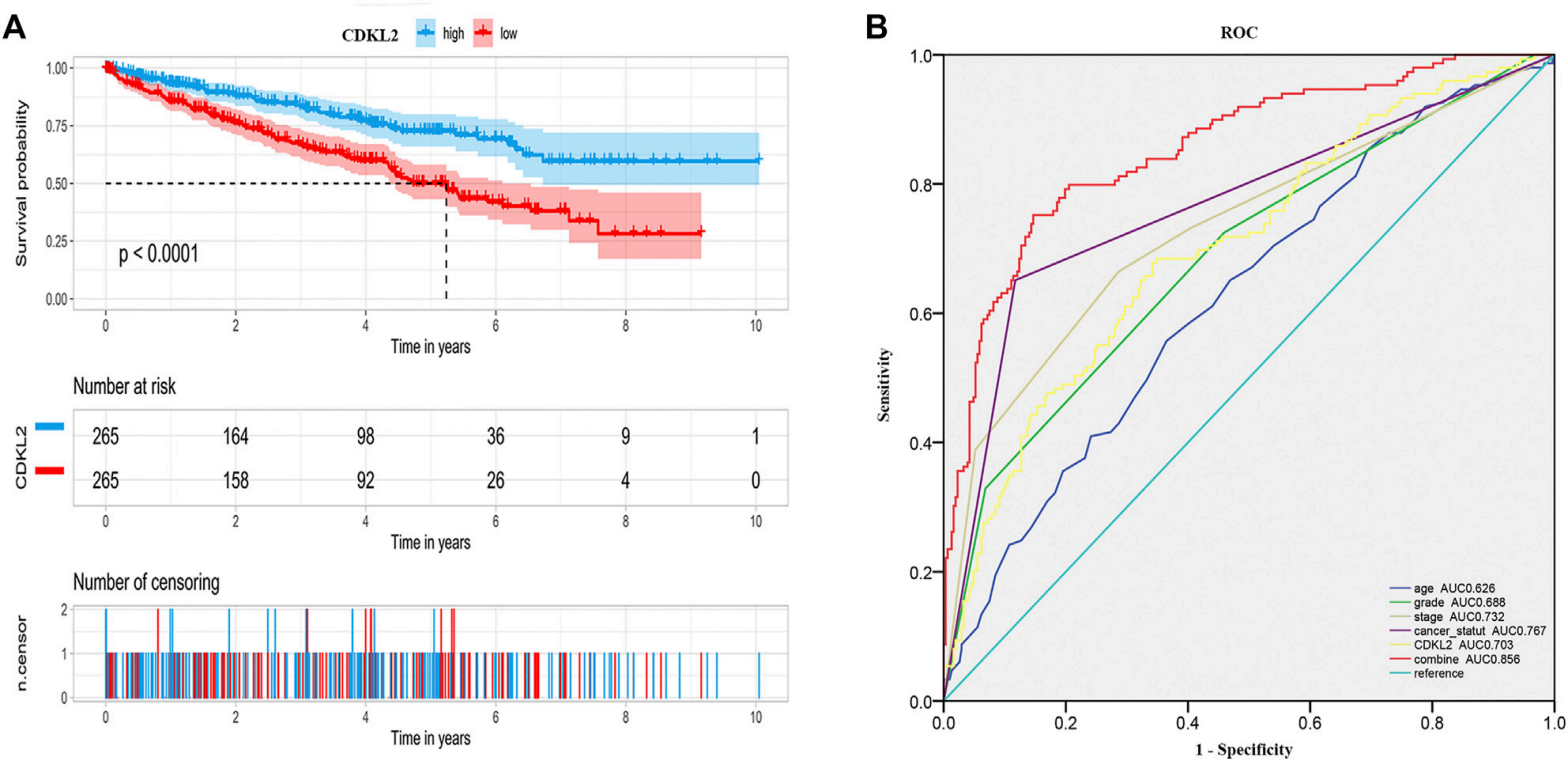
Overall survival of ccRCC patients grouped by CDKL2 median cutoff in TCGA database **(A)**, ROC of overall survival in TCGA-KIRC **(B)**.

**FIGURE 4:**
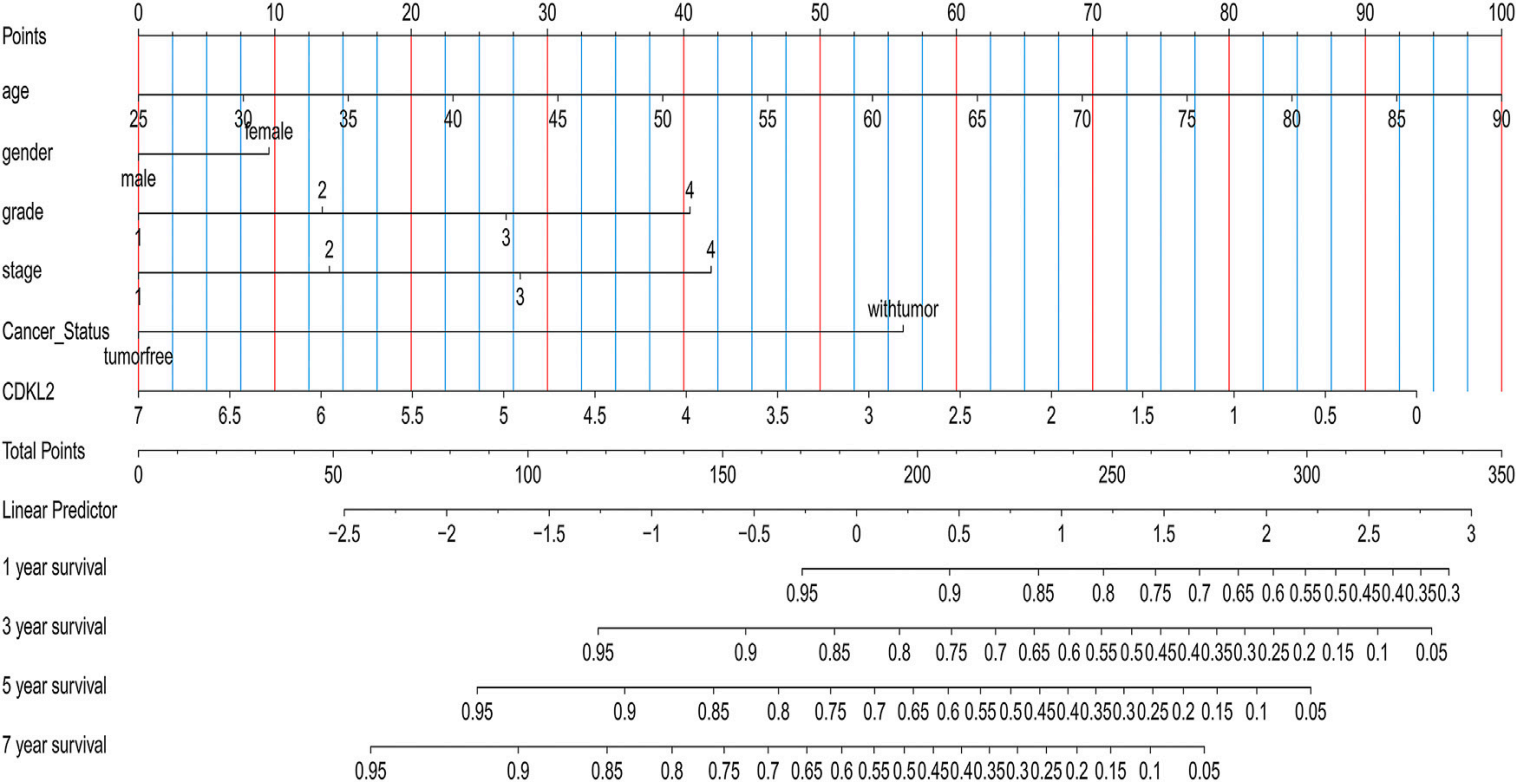
Nomogram for predicting 1-, 3-, 5-,or 7-year survival in ccRCC patients.

We carried out OS analysis of *CDKL2* using “survival” and “survminer” packages in R and the ROC cure was plotted using Kaplan–Meier method. The results indicated that low *CDKL2* expression in ccRCC tissue samples was significantly associated with poor OS (*p* < 0.001; Figure 3A). Further, we performed OS analysis between *CDKL2* mRNA expression and various subgroups of patients with ccRCC (Supplementary Figure S1). Furthermore, univariate Cox analysis revealed that high *CDKL2* expression in ccRCC was closely associated with good OS (HR = 0.502, CI: 0.399–0.630, *p* < 3.217E-09). For multivariate analysis, *CDKL2* correlated independently with OS (HR = 0.764, CI: 0.602–0.970, *p* = 0.027), along with stage and tumor status. The results are shown in Table 3. We generated the ROC curve of *CDKL2*, age, grade, stage and cancer-status, the area under curve (AUC) was 0.703, 0.688, 0.732, 0.767, respectively (Figure 3B). AUC of combine model (*CDKL2* + age + stage + grade + cancer-status) in TCGA was 0.856. The area under ROC curve (AUC) of our *CDKL2* model for overall survival suggested our model had a favorable efficiency in predicting overall prognosis. Predictive model with nomograms integrating age, gender, grade, stage, cancer status, and *CDKL2* expression level in TCGA dataset was generated (Figure 4); the c-index was 0.787. To assess nomogram-predicted 1-, 3-, 5-, and 7-years survival, in Supplementary Figure S2, calibration curves were generated to indicate a good agreement between the nomogram prediction and actual survival.

**TABLE 3:** Univariate analysis and multivariate analysis of the correlation of CDKL2 expression with OS among ccRCC patients.

Parameter	Univariate analysis			Multivariate analysis		
	HR	95% CI	P	HR	95% CI	P
Age (continuous)	1.033	1.018–1.047	5.585E-06	1.033	1.018–1.050	2.390E-05
Gender	0.935	0.668–1.311	0.698			
Stage	1.882	1.632–2.169	2.870E-18	1.616	1.013–2.581	0.044
Histologic grade	2.239	1.800–2.785	4.500E-13	1.324	1.034–1.695	0.026
T classification	1.871	1.570–2.230	2.470E-12	0.732	0.481–1.114	0.145
N classification (N0 + NX *vs.* N1)	3.271	1.600–6.686	0.001	1.484	0.696–3.163	0.307
M classification	4.508	3.228–6.295	9.560E-19	0.922	0.460–1.849	0.820
Cancer Status	5.227	3.729–7.327	8.140E-22	2.676	1.767–4.053	3.33E-06
CDKL2 expression	0.502	0.399–0.630	3.220E-09	0.764	0.602–0.970	0.027

### Co-Expression Analysis of *CDKL2* and GO Enrichment Analysis

**FIGURE 5:**
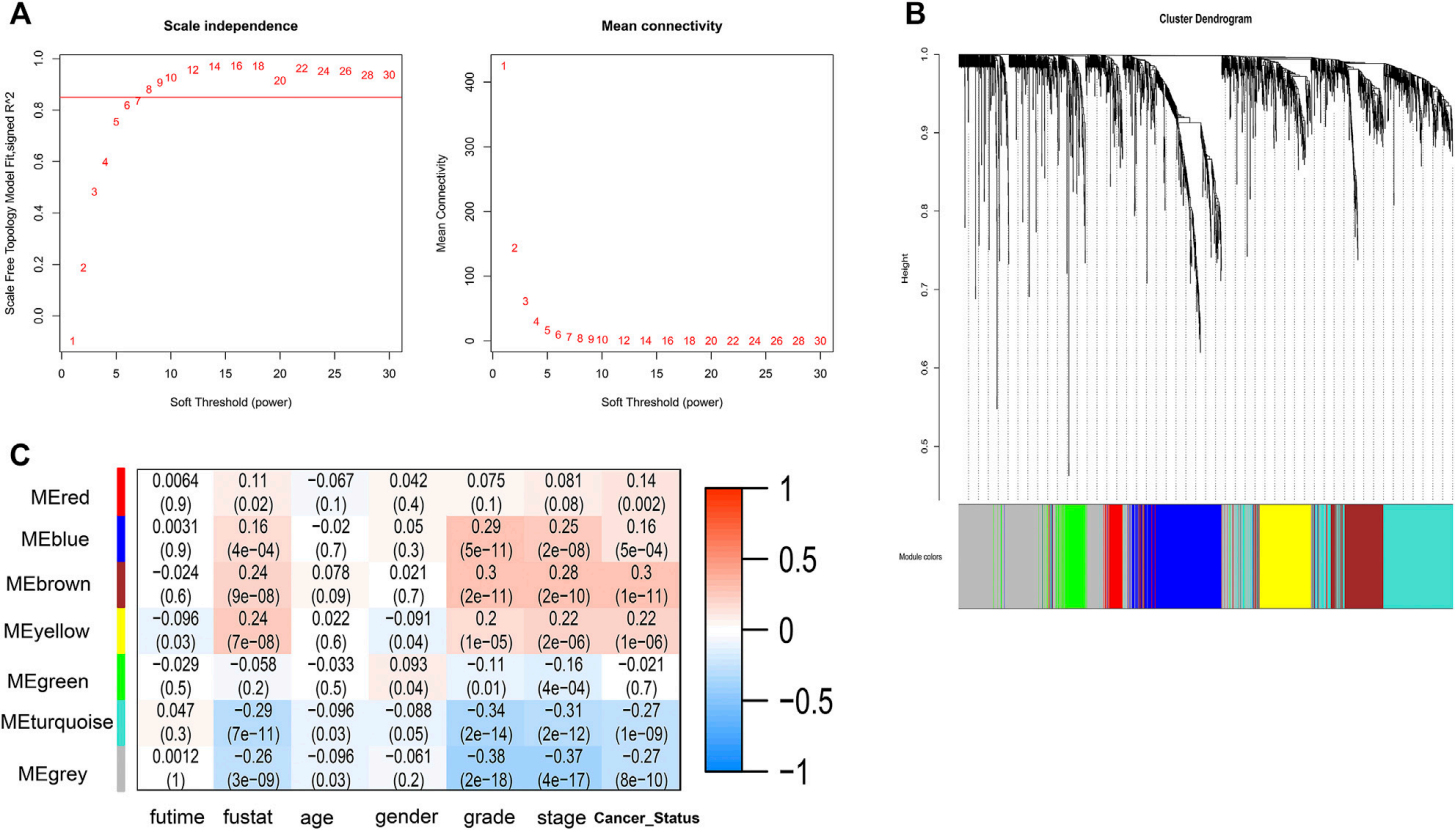
WGCNA network and module detection. **(A)** Selection of the soft-thresholding powers. Power 8 was chosen because the fit index curve flattened out upon reaching a high value (>0.85). **(B)** Cluster dendrogram and module assignment for modules from WGCNA. **(C)** A correlation heatmap between module eigengenes and clinical factors of ccRCC.

A total of 2000 correlated genes (top 1,000 positively and negatively) were subjected to construct a weighted co-expression network using WGCNA; 486 patients with complete clinical information were selected. According to the scale-free network standard, the soft threshold power value was set as 8 (Figure 5A). Blue (353 genes), brown (243 genes), green (135 genes), gray (577 genes), red (74 genes), turquoise (407 genes), and yellow (211 genes) modules were established (Figure 5B). As the module-trait correlation analysis shown in Figure 5C, the blue, brown, and turquoise modules were highly related to grade and stage (*p* < 0.05). Moreover, the brown and turquoise modules were related to cancer status, and the brown, yellow, turquoise modules was significantly related to survival status. Further, we performed GO enrichment analysis of the genes in the hub module. GO terms in BP results are shown in Table 4. Many genes were enriched in “SRP-dependent cotranslational protein targeting to membrane,” “NIK/NF-kappa B signaling,” “cell cycle arrest,” “apoptotic process,” “regulation of immune response,” and “T cell receptor signaling pathway,” etc. As shown in Table 5, genes were related to “Ribosome,” “Endocytosis,” “Pathways in cancer,” and “Natural killer cell mediated cytotoxicity,” in the KEGG pathway. By using Oncobox, we explored the 2000 *CDKL2-*relared gene (top 1,000 positively and negatively) in TCGA database to investigate their major pathway. Kidney 8.0 as the control group, and explore pathway in Biocarta (v1.2), KEGG (v1.2), KEGG (adjust1.4), Qiagen (v1.4), and Reactome (v1.3). In Biocarta, the top five pathway sort by *p*-value were “how does salmonella hijack a cell,” “how does salmonella hijack a cell (lamellipodium assembly),” “role of pi3k subunit p85 in regulation of actin organization and cell migration,” “y-branching of actin filaments,” and “role of pi3k subunit p85 in regulation of actin organization and cell migration (filopodium formation)”. In KEGG (v1.2), the top5 were “Pathways in cancer,” “mRNA surveillance,” “HIF-1_signaling,” “ErbB signaling,” and “Small cell lung cancer”. In KEGG (adjust1.4), the top five was “Protein export,” “Pathways in cancer”, “Proteasome,” “mRNA surveillance,” and “HIF_1_signaling”. The top5 pathway sort by *p*-value in Qiagen (v1.4) were “PTEN Pathway Cell Survival,” “mTOR Pathway Translation Elongation,” “PTEN Pathway Protein Synthesis,” “mTOR Pathway Autophagy,” “mTOR Pathway Mitochondria Proliferation and Function” and in Reactome (v1.3) were “Inactivation of Cdc42 and Rac,” “Bicarbonate transporters,” “HSF1 dependent transactivation,” “Activation of BIM and translocation to mitochondria,” and “Formation of the ternary complex and subsequently the 43S complex”.

**TABLE 4:** Biological processes analysis of genes in the significant module traits in WCGNA and the top100 genes related to CDKL2 methylation.

WGCNA-module	Term	Count	P value
Brown	GO:0006614∼SRP-dependent cotranslational protein targeting to membrane	28	1.88E-30
	GO:0000184∼nuclear-transcribed mRNA catabolic process, nonsense-mediated decay	29	7.42E-29
	GO:0006364∼rRNA processing	35	8.49E-29
	GO:0019083∼viral transcription	28	3.66E-28
	GO:0006413∼translational initiation	28	1.27E-25
	GO:0006412∼translation	34	4.10E-25
	GO:0038061∼NIK/NF-kappaB signaling	7	1.26E-04
	GO:0002181∼cytoplasmic translation	5	1.95E-04
	GO:0010803∼regulation of tumor necrosis factor-mediated signaling pathway	5	4.04E-04
	GO:0043123∼positive regulation of I-kappaB kinase/NF-kappaB signaling	9	6.55E-04
Turoquise	GO:0006886∼intracellular protein transport	17	2.03E-05
	GO:0030148∼sphingolipid biosynthetic process	7	3.54E-04
	GO:0016579∼protein deubiquitination	9	7.87E-04
	GO:0007050∼cell cycle arrest	10	0.002017
	GO:0016567∼protein ubiquitination	17	0.002273
	GO:0006686∼sphingomyelin biosynthetic process	3	0.003769
	GO:0009083∼branched-chain amino acid catabolic process	4	0.005916
	GO:0006897∼endocytosis	9	0.006564
	GO:0016192∼vesicle-mediated transport	9	0.010977
	GO:0016310∼phosphorylation	7	0.01461
Yellow	GO:0070059∼intrinsic apoptotic signaling pathway in response to endoplasmic reticulum stress	5	3.37E-04
	GO:0007249∼I-kappaB kinase/NF-kappaB signaling	6	3.59E-04
	GO:0061154∼endothelial tube morphogenesis	3	0.001505
	GO:0097193∼intrinsic apoptotic signaling pathway	4	0.003442
	GO:0035023∼regulation of Rho protein signal transduction	5	0.009405
	GO:0043525∼positive regulation of neuron apoptotic process	4	0.009501
	GO:0006915∼apoptotic process	13	0.013392
	GO:0007266∼Rho protein signal transduction	4	0.014331
	GO:0032481∼positive regulation of type I interferon production	4	0.015115
	GO:0034097∼response to cytokine	4	0.015922
Blue	GO:0050776∼regulation of immune response	33	3.32E-23
	GO:0006955∼immune response	46	1.43E-22
	GO:0045087∼innate immune response	42	8.53E-19
	GO:0002479∼antigen processing and presentation of exogenous peptide antigen *via* MHC class I, TAP-dependent	14	6.31E-11
	GO:0050852∼T cell receptor signaling pathway	19	1.41E-10
	GO:0006954∼inflammatory response	28	9.18E-10
	GO:0002250∼adaptive immune response	18	1.14E-09
	GO:0007165∼signal transduction	52	1.46E-09
	GO:0060333∼interferon-gamma-mediated signaling pathway	13	3.94E-09
	GO:0042102∼positive regulation of T cell proliferation	12	7.24E-09
Methylation	Term	Count	P value
	GO:0006955∼immune response	8	0.003151
	GO:0007165∼signal transduction	13	0.00634
	GO:0051260∼protein homooligomerization	5	0.008887
	GO:0010871∼negative regulation of receptor biosynthetic process	2	0.013694
	GO:0051496∼positive regulation of stress fiber assembly	3	0.015873
	GO:0030838∼positive regulation of actin filament polymerization	3	0.018091
	GO:0007320∼insemination	2	0.018218
	GO:0006533∼aspartate catabolic process	2	0.018218
	GO:0042752∼regulation of circadian rhythm	3	0.021238
	GO:0006531∼aspartate metabolic process	2	0.027204
	GO:0043401∼steroid hormone mediated signaling pathway	3	0.028155
	GO:0007015∼actin filament organization	3	0.043159
	GO:0006884∼cell volume homeostasis	2	0.044932
	GO:0031295∼T cell costimulation	3	0.049829

**TABLE 5:** KEGG analysis of genes in the significant module traits in WCGNA and the top100 genes related to CDKL2 methylation.

WGCNA-module	Term	Count	P value
Brown	hsa03010:Ribosome	32	3.66E-27
	hsa04141:Protein processing in endoplasmic reticulum	8	0.026833
	hsa00230:Purine metabolism	8	0.032495
	hsa03050:Proteasome	4	0.04
Turoquise	hsa04144:Endocytosis	13	0.00192
	hsa01130:Biosynthesis of antibiotics	11	0.006649
Yellow	hsa04722:Neurotrophin signaling pathway	7	0.00152
	hsa05169:Epstein-Barr virus infection	7	0.001654
	hsa05168:Herpes simplex infection	7	0.011935
	hsa04330:Notch signaling pathway	4	0.013581
	hsa04010:MAPK signaling pathway	8	0.01612
	hsa05203:Viral carcinogenesis	7	0.019898
	hsa05200:Pathways in cancer	10	0.020639
	hsa04622:RIG-I-like receptor signaling pathway	4	0.036543
	hsa04380:Osteoclast differentiation	5	0.047887
Blue	hsa05150:*Staphylococcus aureus* infection	14	3.21E-10
	hsa05416:Viral myocarditis	14	6.65E-10
	hsa04940:Type I diabetes mellitus	12	2.95E-09
	hsa05332:Graft-versus-host disease	11	3.29E-09
	hsa05330:Allograft rejection	11	1.14E-08
	hsa04650:Natural killer cell mediated cytotoxicity	17	3.42E-08
	hsa04380:Osteoclast differentiation	17	9.50E-08
	hsa04145:Phagosome	18	1.13E-07
	hsa04612:Antigen processing and presentation	13	2.43E-07
	hsa04514:Cell adhesion molecules (CAMs)	17	2.95E-07
Methylation	hsa05323:Rheumatoid arthritis	4	0.011487

### Analysis of *CDKL2* Methylation in ccRCC and Related Genes GO Enrichment Analysis

First, we found that *CDKL2* methylation level in ccRCC tissue samples was higher than that in normal tissue samples on screening against the UALCAN database (Figure 1D). Next, we used LinkedOmics to understand the co-expression genes associated with *CDKL2 and its* methylation related genes, as shown in the volcano maps in Supplementary Figures S3A,D. The top 50 genes positively and negatively correlated with *CDKL2* in KIRC were presented in two heatmap (Supplementary Figures S3B,C). Another two heatmaps (Supplementary Figures S3E,F) show the top 50 significant genes that were positively/negatively correlated with *CDKL2* methylation. Further, we explored GO enrichment analysis of the genes and found that the most significant terms in BP were “immune response,” “signal transduction,” and “protein homooligomerization.” The most highly enriched terms from KEGG analysis were “Rheumatoid arthritis.” Finally, we observed the methylation site of *CDKL2* in the MethSurv network, wherein we found that cg00977384, cg14988503, cg05426966, cg14263942, cg10344081, cg03757145, cg20463808, cg24432073, and cg05982271 were significantly related to prognosis. (Table 6).

**TABLE 6:** The significant prognostic value of CpG sites in CDKL2 DNA methylation.

Relation to island	Genomic region	CpG site	HR	LR test p value
Open-sea	Body	cg00977384	0.456	0.00019
Open-sea	3“UTR	cg00859350		0.064
S_Shore	TSS1500	cg05426966	2.31	0.0013
N-shelf	5UTR	cg20463808	1.665	0.015
N_shore	5UTR	cg10131286		0.18
Island	5UTR, 1stExon	cg14988503	0.478	0.00021
Island	TSS200	cg14263942	0.504	0.0014
Island	TSS200	cg10344081	0.554	0.0068
Island	TSS200	cg03757145	0.549	0.0079
Island	5UTR, 1stExon	cg24432073	0.572	0.02
Island	TSS1500	cg05982271	0.613	0.04
Island	TSS1500	cg25060172		0.089
Island	TSS200	cg26173997		0.097
Island	TSS1500	cg02675308		0.11
Island	TSS200	cg21195185		0.17
Island	TSS200	cg02466113		0.79

### GSEA Identifies *CDKL2*-Related Signaling Pathway

**FIGURE 6:**
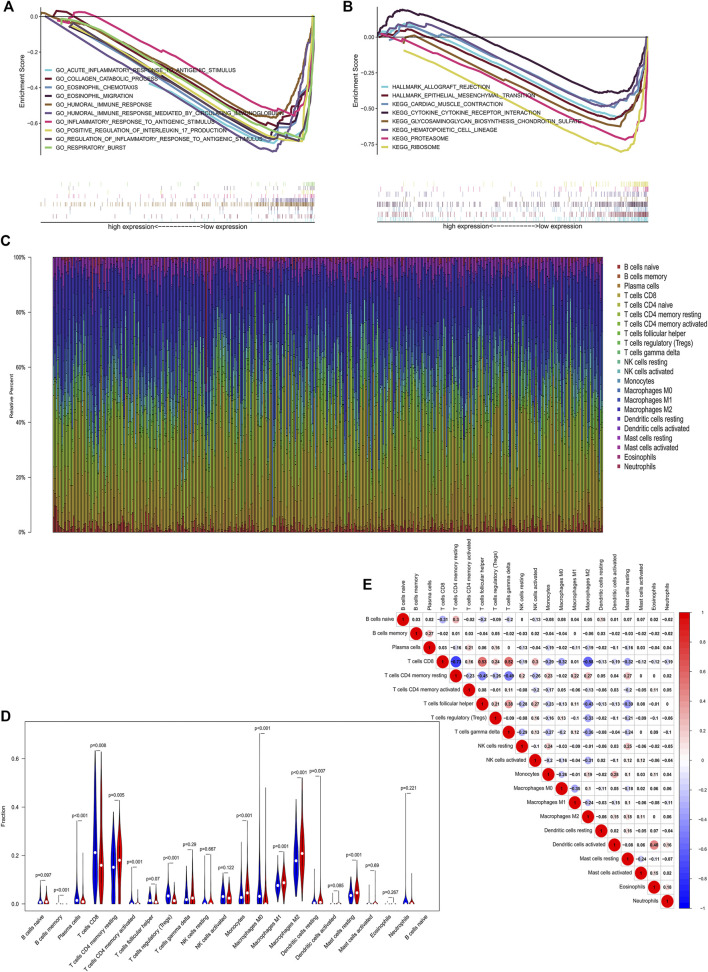
GSEA **(A, B)** and immune cell infiltration analysis **(C–E)**. Enriched pathways in the Low-CDKL2 group based on GSEA. **(A)** Biological processes **(B)** Hallmark and KEGG pathway analysis. Correlations of CDKL2 expression with immune infiltration levels in the TCGA cohort. **(C)** Stacked bar chart representing deviations in immune infiltration in each sample. **(D)** Difference in proportion of each immune cell in low-CDKL2 and high-CDKL2 according to the CDKL2 expressed median value. Blue represents low-CDKL2 samples and red represents high-CDKL2 samples. **(E)** Correlation matrix of immune cell proportions. The red color represents positive correlation and the blue color represents negative correlation.

In order to explore potential signaling pathways activated in ccRCC, we conducted GSEA between the high and low *CDKL2* expression groups. To investigate potential biological processes of CDKL2, “bp V7.1” was used to perform the BP ontology analysis. In the group with low CDKL2, top 10 of gene sets that correlated were “acute inflammatory response to antigenic stimulus,” “collagen catabolic process,” “eosinophil chemotaxis,” “humoral immune response,” “eosinophil migration,” “humoral immune response mediated by circulating immunoglobulin,” “inflammatory response to antigenic stimulus,” “positive regulation to interleukin 17 production,” “regulation of inflammatory response to antigenic stimulus,” and “respiratory burst.” Gene sets associated with allograft rejection “allograft rejection,” “epithelial-mesenchymal transition,” “cardiac muscle condition,” “cytokine-cytokine receptor interaction,” “glycosaminoglycan biosynthesis chondroitin sulfate,” “hematopoietic cell lineage,” “proteasome,” and “ribosome” were differentially enriched in the *CDKL2* low-expressed phenotype in KEGG and Hallmark (hallmark, v7.1) pathway analyses. Details are shown in Figures 6A,B. In summary, these results revealed that CDKL2 was closely associated with immune response.

### Tumor-Infiltrating Immune Cells

It has been previously reported that TIICs can modulate cancer progression and have an effect on survival rate (Singh et al., 2017). Infiltrating CD4^+^ T cells fascinate *TGFβ1* expression and regulated kidney cancer cell proliferation by activating *TGFβ1/YBX1/HIF2α* signals (Wang et al., 2018). We investigated the relationship between *CDKL2* expression and TIICs in ccRCC using the CIBERSORT method. A total of 379 tumor samples from TCGA-KIRC gene expression profiles were used to identify the subpopulation of 22 immune cells. The landscape of immune infiltrations in ccRCC provided from the three normal samples and 379 tumor samples is summarized in Figure 6C. *CDKL2* samples were segregated into high (N = 190) and low (N = 189) expression groups. Proportions of the 22 immune cell subpopulations were generated from CIBERSORT results. As shown in Figure 6D, “T cells CD4 memory resting,” “Monocytes,” “Macrophages M1,” “Dendritic cells resting,” “Macrophages M2,” and “Mast cells resting” were greatly increased in the high expression group. Meanwhile, “Plasma cells,” “T cells CD8,” “T cells regulatory (Tregs),” and “Macrophages M0” were decreased in the high expression group as compared to the low expression group. Then, the proportions of 22 TIIC subpopulations had weak to strong correlations in ccRCC tissues (Figure 6E). “T cells CD8” and “T cells follicular helper” had the strongest positive correlation (cor = 0.53), while “T cells CD8” and “T cells CD4 memory resting” showed the strongest negative correlation (cor = −0.73). Moreover, “T cells CD8” had obviously positive correlation with “T cells gamma delta” (cor = 0.52) and negative correlation with “Macrophages M2” (cor = −0.58). In summary, our findings suggest that *CDKL2* plays a significant role in the immune response in ccRCC.

### Validation in the ICGC, HPA, and GEO Database

**FIGURE 7:**
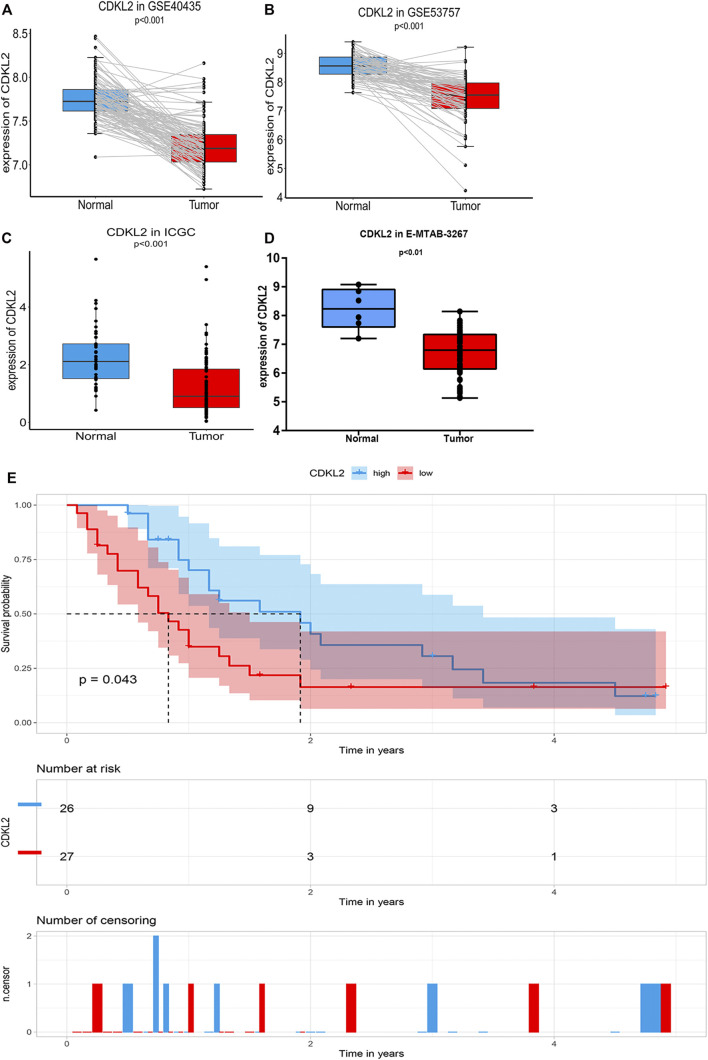
Verification CDKL2 expression in ccRCC tissue and normal kidney tissues. [Figure **(A,B)**] Differential CDKL2 mRNA expression between ccRCC and its’ matched normal kidney tissues in the GSE53757 **[(** Figure 7A, N = 72**)** and GSE40435 **(** Figure 7B, N = 101**)**, respectively. **(C)** Differential CDKL2 mRNA expression between normal tissues and ccRCC in ICGC database [Figure **(C)**] and E-MTAB-3267. [Figure **(D)**]. Figure **(E)** indicate Kaplan–Meier survival (PFS) analysis of E-MTAB-3267.

A total of 101 ccRCC and their matched adjacent normal kidney tissue samples in GSE40435 and 72 paired samples in GSE53757 were found to be differentially expressed using paired Student’s t-test. In ICGC, *CDKL2* expression in ccRCC samples (N = 91) was downregulated compared to that in normal kidney tissue samples (N = 45). At the mRNA level, *CDKL2* was significantly decreased in ccRCC tissue samples as compared to the normal kidney tissue samples in GSE53757, GSE40435, and ICGC (Figures 7A–C). Further, at the protein level, *CDKL2* is decreased in KIRC tissues as compared to that in normal tissues in the HPA (Supplementary Figure S4). In E-MTAB-3267 datasets, *CDKL2* has a significant difference in Normal (N = 6) *vs.* ccRCC (N = 53), *p* < 0.01, was downregulated in ccRCC, the result was consistent with the GEO, ICGC, and TCGA-KIRC datasets. According to the expression level of *CDKL2*, the ccRCC samples (N = 53) were divided into low *CDKL2* (N = 27), and high *CDKL2* (N = 26) groups, Figure 7D. Survival curves were generated using the Kaplan–Meier method, as a result, the PFS survival was generated from the E-MTAB-3267 dataset, as shown in Figure 7E, and Log-Rank *p* < 0.05. From this result, it can be concluded that the survival rate of samples with low *CDKL2* is lower than that of samples with high *CDKL2* in the ccRCC. In addition, these results are consistent with previous results in TCGA-KIRC.

## Discussion

Cell cycle is regulated at the G1/S and G2/M checkpoints by proteins, such as cyclin-dependent kinases (CDKs) and cyclins (Lundberg and Weinberg, 1999). CDKL family is considered as a separate branch of the CDK family, identified by biochemical and genetic methods (Malumbres et al., 1996; Malumbres et al., 2009). CDKL2 is a member of a large family of CDC2-related serine/threonine protein kinases. Although several studies till date have revealed that CDKL2 is essential for tumorigenesis and development, its role in cancer seems to be intricate without deep understanding. To date, only one published study by Li and colleagues (Li et al., 2014) revealed the involvement of *CDKL2* in cancers, wherein they reported that expression of *CDKL2* was remarkably higher in human breast cancer tissues and cells than that in their normal counterparts. In human invasive breast cancers, overexpression of *CDKL2* had a significantly shorter OS time. In addition, *CDKL2* promotes epithelial-mesenchymal transition and increases CD44-high mesenchymal cell subpopulation by upregulating *ZEB1* expression. Their results suggested that *CDKL2* serves as a carcinogenic gene in breast cancer, promoting epithelial-mesenchymal transition and tumor progression. However, the detailed data from their research are not coherent with those described in the Oncomine database. Further, Lindqvist et al. (2014) reported that *CDKL2* was hypermethylated in tumors and its expression was downregulated in HER2+ breast cancer tissues compared to the normal tissues. Similar to most studies, *CDKL2* was considered a tumor suppressor gene and its upregulated expression was suggested to inhibit cancer cell proliferation and invasion. In gastric cancer (Fang et al., 2018), loss of *CDKL2* expression was significantly correlated with clinicopathological characteristics (such as pathologic staging, histologic type, and grade); moreover, it could shorten patient disease-free survival and OS. Likewise, a study that included 151 glioma and 34 para-carcinoma tissues was analyzed by qPCR, western blot, and immunohistochemistry. They reported that the expression of *CDKL2* in glioma tissues was significantly lower than that in non-cancerous brain tissues (Yi et al., 2020). In addition, decreased *CDKL2* expression predicted an evidently poor OS in glioma. Further, Zhou et al. (2019) investigated *CDKL2* expression levels in 178 HCC, 169 adjacent non-cancerous, and 24 normal liver tissues. They reported that compared to normal liver tissues, *CDKL2* mRNA expression was downregulated in HCC cell lines and tumor tissues, which was inversely related to DNA methylation, suggesting that *CDKL2* methylation may be involved in tumorigenesis and progression. Besides, *CDKL2* expression can be upregulated by treating HCC cell lines with 5-aza-2-deoxycytidine. Rubicz et al. (2019) reported a high methylation level in the promoter region of *CDKL2* and a corresponding decrease in mRNA levels in patients with more aggressive prostate cancer. Zhao et al. (2019) found that in HeLa cells, HSV-2- encoded miRNA-H4 regulates cell cycle progression and actinomycin D. Further, targeting *CDKL2* and *CDKN2A* induced apoptosis in these cells. In the recent time, Ruan et al. (2020) constructed a renal clear cell carcinoma model containing CDKL2, LRFN1, STAT2 and SOWAHB in KIRC, and also verified that CDKL2 is low in renal clear cell carcinoma and is associated with prognosis, but there is no further research on CDKL2 related methylation site, GO enrichment analysis of related genes, clinical application of nomogram, and potential mechanism related to immunity.

In this study, we performed a series of bioinformatic analyses on the publicly available ccRCC database for elucidating the expression of *CDKL2*. First, we identified that *CDKL2* mRNA expression levels were notably decreased in ccRCC tissues as compared to normal kidney tissues. This result was in concurrence with the abovementioned cases of gastric cancer, HCC, and glioma. Further, we studied the correlation between *CDKL2* and clinicopathological features. In this subgroup analysis, *CDKL2* downregulation was associated with certain clinicopathological factors including gender, poor tumor grade, advanced TNM stages, positive nodal invasion, metastasis, tumor status, and survival of patients with ccRCC. We found that decreased *CDKL2* expression was linked to poor prognosis. By generating ROC curves, we found that *CDKL2* could be a prognostic marker for the OS of patients with ccRCC. At the same time, we obtained *CDKL2* as an independent prognostic factor for ccRCC through Cox analysis. Based on *CDKL2* expression levels and clinical factors, we generated a nomogram to predict the OS rate of patients with ccRCC. To further investigate its mechanism in ccRCC, genes associated with *CDKL2*, including WGCNA and methylation, were analyzed. Functional enrichment analysis showed that *CDKL2* is mainly enriched in immune responses. Based on these results, we suggest that low expression of *CDKL2* may be linked to immunity in ccRCC. Moreover, we simultaneously revealed that multiple immune cell infiltrations were associated with the differential expression of *CDKL2* in ccRCC by CIBERSORT. The result indicated that expression of *CDKL2* is closely correlated with the infiltration levels of a variety of immune cells. Although we used several databases to validate the results that adds merits to the present study, there is a major limitation associated. Briefly, we did not perform experimental research for exploring potential mechanisms of *CDKL2* in ccRCC and their correlation with immune cell infiltrations. Further, our future prospects include not only bioinformatic analyses but *in vitro* and/or *in vivo* experiments as well, to strengthen the findings of the present study.

Our findings may guide us to further investigate the importance/association of *CDKL2* in ccRCC, the potential mechanism of *CDKL2* expression, and immune interaction in tumor development and progression. In addition, *CDKL2* may be a favorable biomarker for diagnosis and treatment of ccRCC in the future.

## Data Availability

Publicly available datasets were analyzed in this study. This data can be found here: TCGA (https://portal.gdc.cancer.gov), ICGC (https://icgc.org/), NCBI Gene Expression Omnibus (GSE40435 and GSE53757), and ArrayExpress (E-MTAB-3267).

## Abbreviations

ccRCC, clear cell renal cell carcinoma; GEO, gene expression omnibus; GO, gene ontology; GSEA, gene set enrichment analysis; HCC, hepatocellular carcinoma; HPA, human protein atlas; ICGC, international cancer genome consortium; KEGG, kyoto encyclopedia of genes and genomes; KIRC, kidney renal clear cell carcinoma; OS, overall survival; RCC, renal cell carcinoma; TCGA, the cancer genome atlas; TIICs, tumor-infiltrating immune cells.
