# Supplementary figures and images for: Decreased CDKL2 Expression in Clear Cell Renal Cell Carcinoma Predicts Worse Overall Survival
Source: Front Mol Biosci. 2022 Jan 13;8:657672. doi: 10.3389/fmolb.2021.657672 (PMC8793634; doi:10.3389/fmolb.2021.657672)

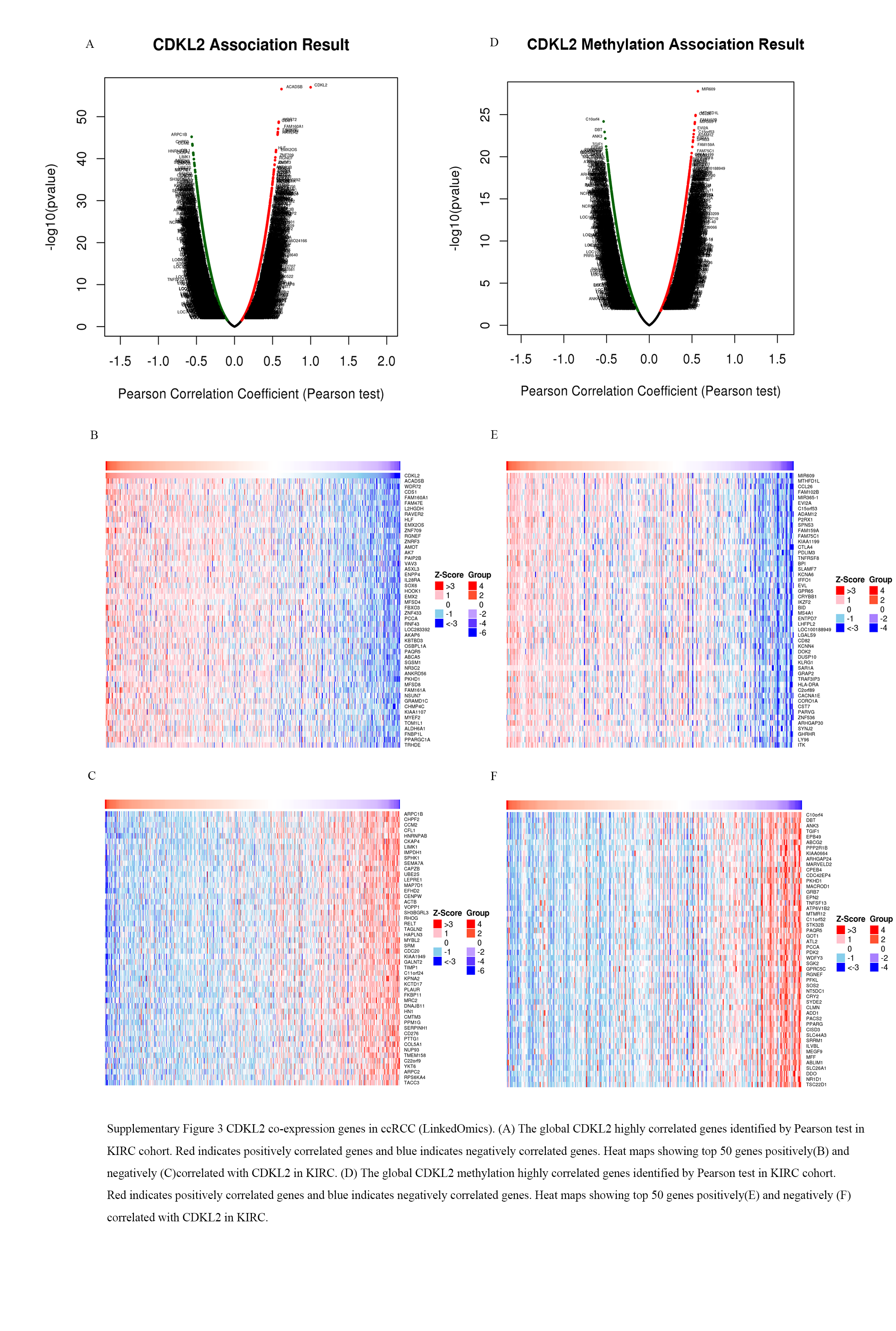

Supplement: Supplementary file 1 [file Image3.TIF]

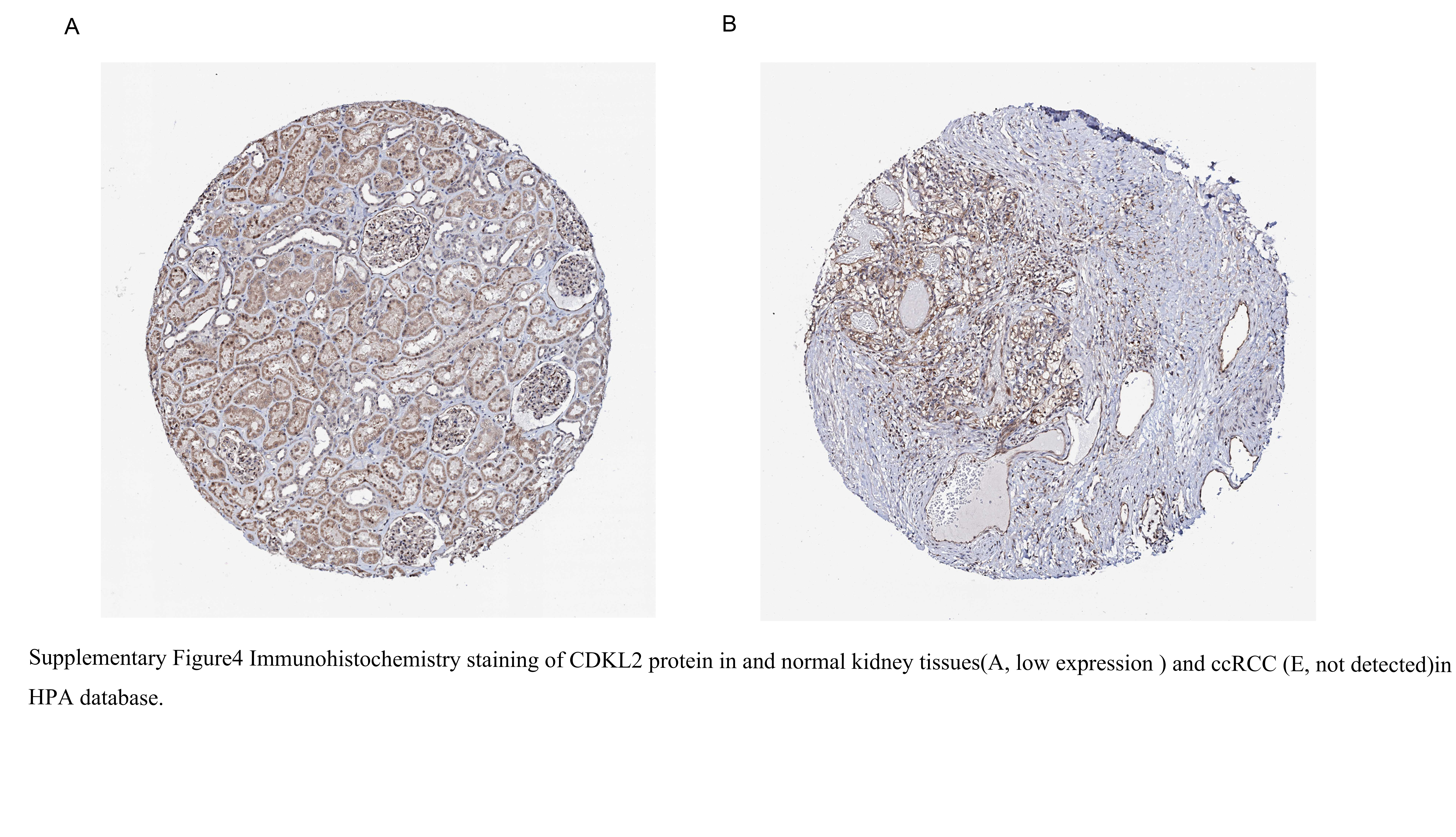

Supplement: Supplementary file 2 [file Image4.JPEG]

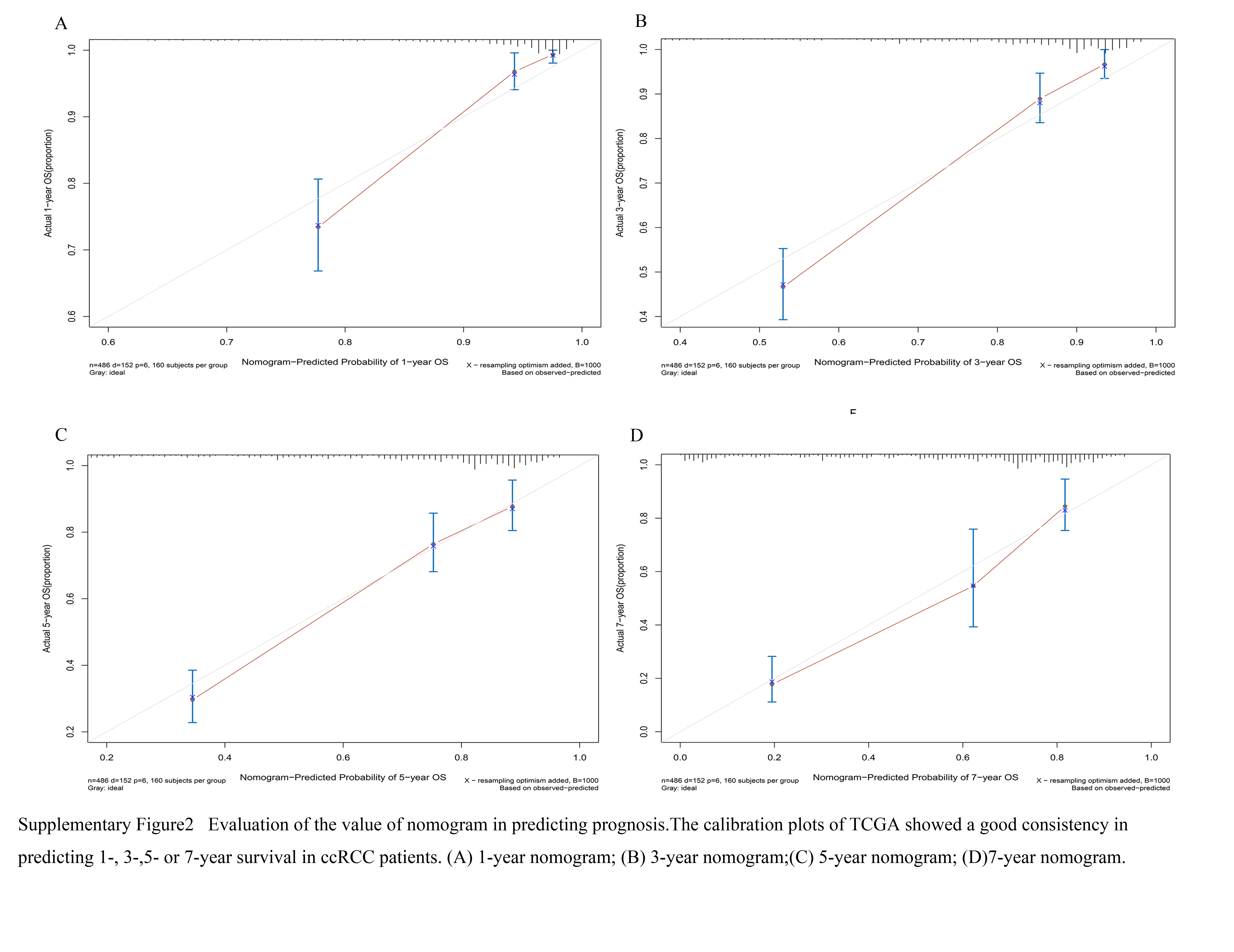

Supplement: Supplementary file 3 [file Image2.TIF]

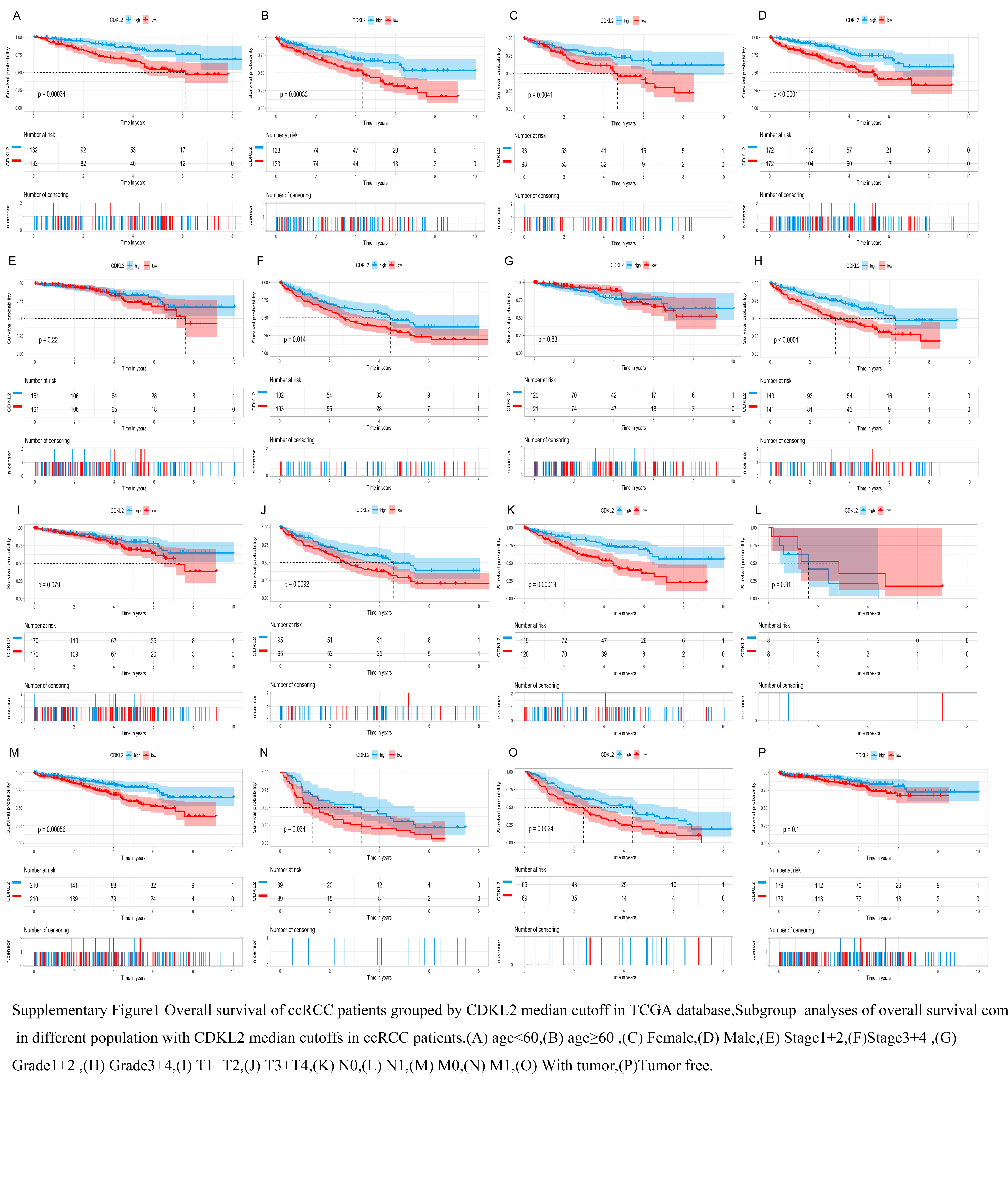

Supplement: Supplementary file 4 [file Image1.TIF]
